# Stress-Driven Production of γ-Aminobutyric Acid Using Non-Conventional Yeast Strains *Kluyveromyces marxianus* JMY140K and *Metschnikowia reukaufii* JMY075

**DOI:** 10.3390/jof11010020

**Published:** 2024-12-31

**Authors:** Ting-Ting Fan, Chao Chen, Du-Wen Zeng, Feng-Lou Wang, Zhao-Xian Xu, Ming-Jie Jin, Yue Zou, Jun Li, Xin-Qing Zhao

**Affiliations:** 1 State Key Laboratory of Microbial Metabolism, Joint International Research Laboratory of Metabolic & Developmental Sciences, School of Life Sciences and Biotechnology, Shanghai Jiao Tong University, Shanghai 200240, China; tingtingfan97@163.com (T.-T.F.); zengduwen@sjtu.edu.cn (D.-W.Z.); 2 College of Life Science, Dalian Minzu University, Dalian 116600, China; chaochen@dlnu.edu.cn; 3 R&D Center, Shanghai CHANDO Group Co., Ltd., Shanghai 200233, China; wangfenglou@chandogroup.com (F.-L.W.); zouyue3@chandogroup.com (Y.Z.); 4 School of Environmental and Biological Engineering, Nanjing University of Science and Technology, Nanjing 210094, China; xzx018@njust.edu.cn (Z.-X.X.); jinmingjie@njust.edu.cn (M.-J.J.); 5 Himalaya Third Pore (Shanghai) Biotechnology Co., Ltd., Shanghai 201499, China

**Keywords:** non-conventional yeast, *K. marxianus*, *M. reukaufii*, γ-aminobutyric acid production, stress stimulation

## Abstract

γ-Aminobutyric acid (GABA) is a valuable amino acid widely used in food, healthcare, and agriculture. GABA bioproduction by budding yeasts has been commonly reported, but related studies using non-conventional yeasts remain limited. In this study, two non-conventional natural yeast strains, namely, *Kluyveromyces marxianus* JMY140K and *Metschnikowia reukaufii* JMY075, were identified as promising GABA producers, and *M. reukaufii* JMY075 was discovered to be a GABA producer. Enhanced GABA production was observed in the two yeast strains under stress conditions, including high temperature and high ethanol and acetic acid levels. In particular, *K. marxianus* JMY140K showed 7.93 times higher GABA titers under thermal stress than that of the control. External stress conditions significantly influenced the GABA production of these two yeast strains. The culture filtrate of *K. marxianus* JMY140K also showed promising activities in human skin cells. In addition, *K. marxianus* JMY140K could also produce GABA using rice straw hydrolysate, which indicated that it has the potential to produce GABA using renewable biomass. Our studies provide insight for further enhancing the GABA production of natural yeasts and promoting its biotechnology applications.

## 1. Introduction

γ-Aminobutyric acid (GABA) is a four-carbon non-protein amino acid that occurs naturally in plants [1], animals [2,3], and microorganisms [4,5]. GABA is widely used in food, health products, agriculture, and cosmetics for its many beneficial physiological functions. For example, GABA can enhance the antagonistic efficacy of *Sporidiobolus pararoseus* Y16 [6], which can be used against postharvest diseases affecting grapes [7]. GABA is also an inhibitory neurotransmitter in the human body and mainly regulates synaptic transmission and relaxes nerves, therefore playing an important role in sleep and combatting depression [8]. GABA can act as a skin protection agent in cosmetics [9,10] due to its anti-hypertensive, anti-diabetic, and anti-cancer effects [11]. It can also increase stress resistance in plants [12], and GABA supplementation can improve the growth of *Eriocheir sinensis* [13], indicating that it can be used as a beneficial food additive.

Yeasts are unicellular eukaryotic microorganisms widely used in food, health products, agriculture, and cosmetics. Some yeasts are known to be GABA producers, such as budding yeast *Saccharomyces cerevisiae* [14], *Pichia kudriavzevii* [15], *Sporobolomyces carnicolor* [16], and *Kluyveromyces marxianus* [17]. Non-Saccharomyces yeasts used in the production of vino cotto proved to be good GABA producers and could be used to enhance the functional characteristics and quality of fermented foods and beverages, showing great potential in producing diversified or innovative high-quality fermented foods [18]. Taking advantage of the fact that the culture broth of yeasts can be directly used in cosmetics or as biofertilizers, the applications of yeasts in GABA production are promising for commercialization.

Among various yeasts, *K. marxianus* is a GRAS (generally recognized as safe) yeast certified by the US (The United States) FDA (Food and Drug Administration). Substances added to food require premarket review unless they are generally regarded as safe (GRAS) [19,20]. The GRAS status of microbes benefits their applications in the food and feed industries. In 2013, the National Health and Family Planning Commission of China approved this yeast as an edible strain. A study has shown that several natural strains of *K. marxianus* can use glucose to produce GABA, but the highest concentration is only 7.78 mg/L [17]. In addition, another non-conventional yeast, *Metschnikowia reukaufii*, which is widely present in nectar [21], is a nectarivorous yeast [22] and can produce isomelzitose [21] and D-arabitol [23]. By optimizing medium composition and fermentation conditions, the yield of D-arabitol in *M. reukaufii* was successfully increased [24], constituting a process that has received much attention for its application in food and agricultural biotechnology. However, no reports on GABA production from *M. reukaufii* have been found to date.

In this work, the non-conventional yeast *M. reukaufii* was discovered to be a GABA producer, and another non-conventional yeast strain, *K. marxianus* JMY140K, was also proved to be a promising GABA producer. Furthermore, the effects of different stress conditions on the GABA production of these two strains were well studied. The results showed that the GABA production of the two non-conventional yeasts was significantly increased by setting a high temperature and adding ethanol and acetic acid. In addition, the effects of bioferments of *K. Marxianus* MY140K on human skin cells were also tested. These tests were used to detect the effect of the product on skin cell repair and further speculate on whether the product has potential applications in cosmetics. The results of this study indicate that the fermentation broth of JMY140K has great potential in cosmetic production. Rice straw is abundantly available as an agricultural waste, so converting it into valuable products is not only crucial for sustainable resource management but also environmental protection [25]. In this study, *K. marxianus* JMY140K was also able to produce GABA using rice straw hydrolysate, which indicated that it has the potential to produce GABA for applications in fields besides cosmetics, such as in the agriculture and feed industries. Our study provides further insights into enhancing the GABA production of natural yeasts and promoting the biotechnology applications of this process.

## 2. Materials and Methods

### 2.1. Strains and Media

Two yeast strains, *K. marxianus* JMY140K and *M. reukaufii* JMY075, isolated from Tibet Autonomous Region, China, were used in this study. They were grown on yeast-extract peptone dextrose (YPD) medium (10 g/L of yeast extract, 20 g/L of peptone, and 20 g/L of glucose). Yeast extract, peptone, glucose, and glutamate were purchased from Sangon Biotech, Shanghai, China; pyridoxal 5′-phosphate was purchased from Energy Chemical, Anqing, China. Rice straw hydrolysate was provided by Mingjie Jin of Nanjing University of Science and Technology.

Total DNA was extracted using the following steps: (1) Take 300 μL culture (with an OD_600_ of about 0.4) and centrifuge it at 5000 rpm for 1 min; discard the supernatant. (2) Add 100 μL of 200 mM LiAC-1% SDS solution and resuspend the yeast cells in a water bath at 70 °C for 15 min. (3) Add 300 μL of 100% ethanol to resuspend the yeast cells, centrifuge this mixture at 12,000 rpm for 5 min, and discard the supernatant. (4) Add 200 μL of 70% ethanol to resuspend the yeast cells, centrifuge them at 12,000 rpm for 2 min, discard the supernatant, and repeat this process twice. (5) Dry the cells in a 55 °C oven for 15 min, and then add 100 μL of sterile water to resuspend the precipitate. (6) Centrifuge the product at 12,000 rpm for 1 min, collect the supernatant to obtain yeast total DNA, and store it at −20 °C. LiAC-1% SDS solution, ethanol were purchased from Sangon Biotech, Shanghai, China.

The strains were identified using the 26 s D1/D2 rDNA region amplified with primers F (5′-GCATATCGGTAAGCGGAGGAAAAG-3′) and R (5′-GGTCCGTGTTTCAAGACGG-3′) [26]. The PCR procedure was performed using the method described by Wang et al. (2010) [26]. Sequencing was performed by tsingke biotechnology Co., Ltd., Beijing, China. The sequences were blasted on the NCBI website (https://blast.ncbi.nlm.nih.gov/Blast.cgi, accessed on 10 March 2020). The Genebank numbers of *K. marxianus* Y140K and *M. reukaufii* Y075 were OM279504 and OM279505, respectively.

### 2.2. Strain Fermentation Conditions and Reparation of Y140K Fermentation Filtrate

In a 5 L microbial bioreactor (Bio-Stat A, Sartorius Scientific Instruments (Beijing) Co., Ltd., Beijing, China) with 3 L YPD media, 5% (*v*/*v*) seed cells were inoculated for GABA fermentation. *M. reukaufii* JMY075 was cultured at 25 °C at 200 rpm for 24 h, and *K. marxianus* JMY140K was fermented at 28 °C or 40 °C for 48 h. The cell density of the strains was measured at 600 nm in a 96-well plate using a microplate reader (ThermoFisher Scientific, Waltham, MA, USA). The fermentation broth was centrifuged at 8000 rpm for 10 min. The supernatant was absorbed by certain types of activated carbon in optimal condition, and the processed mixture was centrifuged and filtered through a 0.22 μm nylon filter membrane at 4 °C. The solid content of the fermentation filtrate of *K. marxianus* JMY140K was approximately 10 mg/mL.

### 2.3. Determination of Extracellular GABA Production

The filtrates of JMY140K were diluted 50 times and passed through a 0.22 μm filter membrane, which was used for further GABA detection. The content of GABA was determined using a UPLC-MS (Waters, Milford, MA, USA) mass spectrometer. The mobile phases were as follows: A—0.1% FA (Formic acid)-H_2_O and B—ACN (Acetonitrile). The column (Waters ACQUITY UPLC HSS T3 1.8 μm, 2.1 × 100 mm) was kept at 35 °C, and a 2 μL processed sample was used for detection. The flow rate was 0.25 mL/min, and the retention time of GABA was 1.010 min. GABA content was calculated using a GABA standard curve; GABA standard, ACN, and FA were purchased from Sangon Biotech, Shanghai, China.

### 2.4. Determination of Intracellular GABA Production

After fermentation for 24 or 48 h, 50 mL cultures were centrifuged at 4 °C to collect the cells and washed with sterile double-deionized water, and then 0.1 mol/L hydrochloric acid (GR) solution and glass beads were used to break the cells with a high-throughput tissue grinder (Ningbo Scientz Biotechnology Co., Ltd., Ningbo, China). Then, we collected all the supernatants and added an appropriate amount of sulfosalicylic acid, leaving the supernatants to stew at 4 °C for 1 h, after which they were centrifuged at 4 °C for 30 min. All supernatants were collected. pH was adjusted to 1.9, and then the supernatants were passed through a 0.22 μm filter membrane. The content of GABA was detected using high-speed amino acid analysis. At the same time, the dry weights of the same cells were measured.

### 2.5. Determination of Intracellular ROS

ROS were detected using the Reactive Oxygen Species (ROS) Assay Kit (Beyotime, Shanghai, China). The cell culture was collected and adjusted to OD_600_ = 1, washed twice with PBS buffer, and then resuspended with DCFH-DA (10 μM) and incubated at 37 °C on a shaker for 20 min, turning it up and down every 5 min. The cells were then washed three times with PBS buffer to completely remove the probe. Fluorescence was measured under 488 nm excitation light and 525 nm emission light using a multifunction microplate reader (Spark, China Tecan (Shanghai) laboratory equipment Co., Ltd., Shanghai, China). Data were calculated using the fluorescence and the OD_600_ value.

### 2.6. Determination of Cell Membrane Permeability

The cell culture was collected, adjusted to OD_600_ = 1, washed twice with PBS buffer, and then resuspended in the diluted PI staining solution and incubated in the dark for 20 min, mixing it up and down every 5 min. The cells were then washed twice with PBS buffer. Fluorescence was measured under 513 nm excitation light and 617 nm emission light using a multifunction microplate reader (Spark, China Tecan (Shanghai) laboratory equipment Co., Ltd., Shanghai, China). Data were calculated based on the fluorescence and the OD_600_ value.

### 2.7. Reverse Transcription Quantitative PCR (RT-qPCR)

Cells were collected and washed twice with sterile double-deionized water. Total RNA was extracted using Hipure Yeast RNA Kit (Magen, Guangzhou, China). Then, cDNA was obtained using Goldenstar^TM^ RT6 cDNA Synthesis Kit Ver.2 (Tsingke, Beijing, China). The cDNA was used as the template for qPCR. The total reaction volume of RT-qPCR was 20 µL. And the reactions were performed in a 96-well plate using SYBR Premix Ex Taq™ (Tsingke, Beijing, China). The qPCR reaction was performed using the CFX Connect Real-Time System (Bio-Rad, Hercules, CA, USA).

### 2.8. Determination of Skin Cell Viability

Fibroblasts (FBs) and human scalp dermal papilla (DP) cells were cultured and maintained in MEM/DMEM (Gibco, Waltham, MA, USA) supplemented with 10% fetal bovine serum (FBS) (Gibco, Waltham, MA, USA) at 37 °C with 5% CO_2_. Cells at 80% confluence were trypsinized and plated in a 96-well plate at a density of 5000 cells/well. After confluency for 48 h, the cells were cultured without FBS. After 16 h of starvation, the cells were treated with different concentrations of JMY140K filtrate. The culture medium in which the filtrates of JMY140K were replaced with PBS was used as a blank control. After 24 h of culturing, the viability of non-treated and treated cells was measured and compared using Cell Counting Kit-8 (CCK-8) (Dojindo, Tokyo, Japan). The proliferation rate was measured at 550 nm, and the proliferation effects of the filtrates of JMY140K on FB and DP were evaluated in comparison to the blank control.

### 2.9. Determination of Cell Repair of UV-Damaged Cells

The fermentation products of the filtrates of JMY140K fermented at 28 °C and 40 °C were added to the cultures of human epidermal (HaCaT) and FB cells, respectively. After overnight starvation culture, the culture medium was removed, and PBS was added for UV irradiation (HaCaT with 20 mJ of UVB and FB with 5.0 J of UVA and 50 mJ of UVB). The irradiated cells were then added to a culture medium containing the filtrates of JMY140K and maintained in the culture for 24 h. Cell viability was measured by using the CCK8 method. The non-irradiated group was used as a blank control. The cell viability of the irradiated, untreated blank group was determined to be 100%, and the effect of the sample on the relative viability of skin cells was evaluated.

### 2.10. Determination of Intracellular ATP in Human Skin Cells

Different concentrations of the filtrates of JMY140K fermented at 28 °C and 40 °C were added the cell cultures of FB, HaCaT, and DP cultured for 24 h, respectively. Then, the culture medium was discarded, and 200 μL lysis solution was added to the lysed cells and then centrifuged at 4 °C and 12,000× *g* for 5 min to collect the supernatants. The bioenergetic effect of non-treated and treated cells was evaluated by measuring cellular ATP content using CellTiter-Glo^®^ Reagent (Promega, Madison, WI, USA). Assay procedures were performed according to the protocol provided by the manufacturer.

### 2.11. Analytical Methods

Data were analyzed using Prism 8.0 (GraphPad Software Inc., La Jolla, CA, USA) with one-way analysis of variance (ANOVA). A level of *p* < 0.05 was considered statistically significant.

## 3. Results and Discussion

### 3.1. Basic Characteristics of the Two Strains

**Figure 1:**
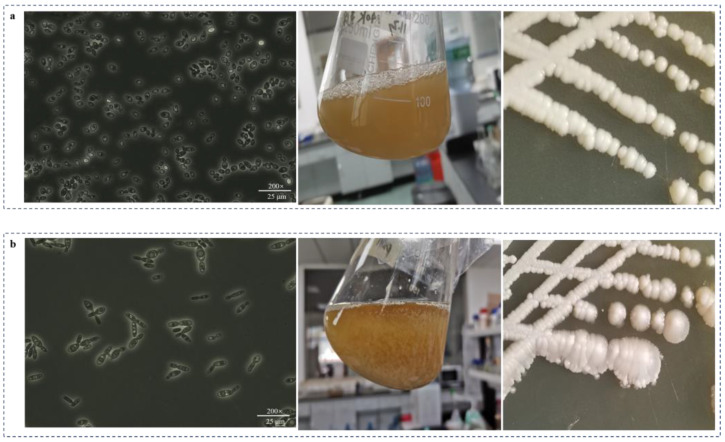
Optical microscope images and growth of the yeast strains *K. marxianus* JMY140K (**a**) and *M. reukaufii* JMY075 (**b**) in YPD liquid and agar medium.

**Figure 2:**
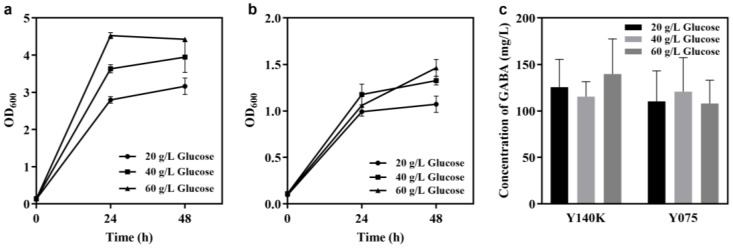
Effects of glucose concentrations on growth and GABA production. (**a**,**b**) Growth of *K. marxianus* JMY140K and *M. reukaufii* JMY075, respectively in YPD liquid media with 20 g/L, 40 g/L, and 60 g/L of glucose, respectively. (**c**) concentration of extracellular GABA produced by Y140K (48 h) and Y075 (24 h). Data are averages from two independent experiments; error bars represent SD.

In this study, two non-conventional GABA-producing yeasts, designated as *K. marxianus* JMY140K and *M. reukaufii* JMY075, respectively, were investigated. Both strains have specific characteristics compared to other yeasts. *K. marxianus* JMY140K has an elliptical shape when viewed under a microscope (Figure 1a). The optimum growth temperature was 30 °C, and the growth rate at 40 °C had obviously decreased. This is different from strains reported in the literature, which have strong high-temperature tolerance and grow faster at 40–45 °C [27]. The optimal growth temperature of *M. reukaufii* JMY075 was 25 °C, and the long spindle was observed under the microscope. It is worth noting that multiple vacuoles were observed in one cell, and JMY075 showed self-flocculation during the fermentation process (Figure 1b). *M. reukaufii* JMY075, which is a nectarivorous yeast [22], was the first example of a GABA producer found in our study, and previous studies showed that it can produce isomelzitose [21] and D-arabitol [23,24], so it has received a great deal of attention for its application in food and agricultural biotechnology. A previous study tested the GABA production ability of 50 strains of *K. marxianus*, and the results indicated that the production of GABA for these 50 strains ranged from 2.54 ± 0.87 mg L^−1^ to 7.78 ± 1.88 mg L^−1^ [17]. In this study, *K. marxianus* JMY140K isolated from Tibet could produce 125.6 mg/L of GABA in the YDP medium (Figure 2c), showing that it is an excellent GABA producer and has great potential for application in food and agricultural biotechnology.

### 3.2. Effects of Glucose Concentration on Extracellular GABA Production

To test the effects of glucose concentration on GABA production, three glucose concentrations, 20, 40, and 60 g/L, were used as carbon sources. The results showed that higher glucose concentrations did not lead to higher extracellular GABA production. For strain JMY140K, the growth rate and extracellular GABA production levels were highest when the glucose concentration was 60 g/L (Figure 2a,c), indicating that for JMY140K, the higher the sugar concentration, the better the growth rate and extracellular GABA production. However, for strain JMY075, it first grew faster with a 40 g/L glucose concentration than a 60 g/L glucose concentration over 24 h, and then it was observed to have grown faster with a 60 g/L glucose concentration at the 48 h mark (Figure 2b). Thus, there was an initial growth inhibition of JMY075 with a 60 g/L glucose concentration. Extracellular GABA production was detected under different glucose concentrations (Figure 2c), which indicated that there were no significant differences in the extracellular GABA production, so a glucose concentration of 20 g/L was used for further GABA fermentation.

### 3.3. Effects of Fermentation Stresses on GABA Production

#### 3.3.1. Growth Rates and GABA Production of Two Strains at Different Temperatures

**Figure 3:**
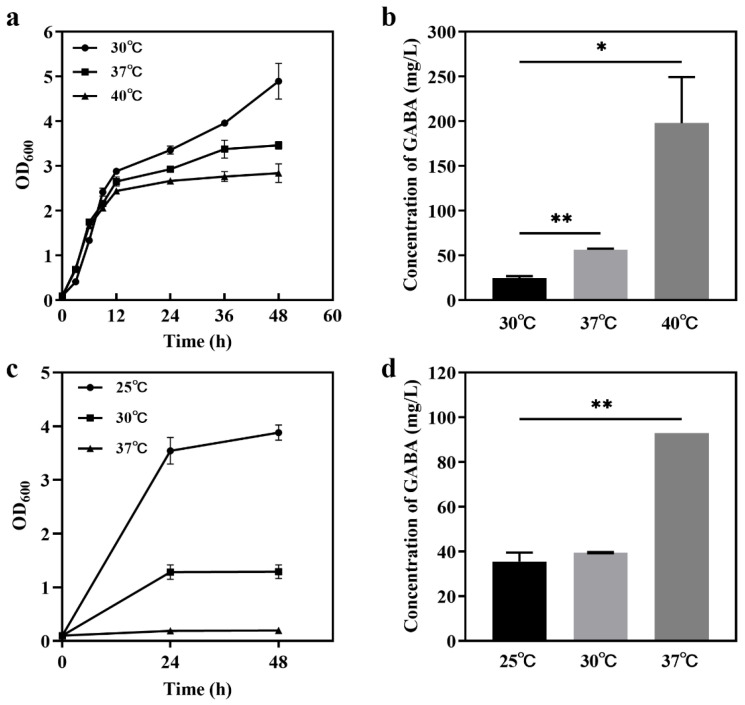
Growth and extracellular GABA production of *K. marxianus* Y140K and *M. reukaufii* Y075 at different temperatures. (**a**,**c**) Growth of Y140K (**a**) and Y075 (**c**) in YPD liquid media; (**b**,**d**) the concentrations of extracellular GABA produced by Y140K (**b**) and Y075 (**d**) in YPD liquid media at 48 h. *, *p*-value < 0.05; **, *p*-value < 0.01.

Temperature is an important factor in the fermentation and production of natural compounds for yeasts. In this study, three temperatures in two groups, 30 °C, 37 °C, and 40 °C and 25 °C, 30 °C, and 37 °C, were used to detect the production of extracellular GABA produced by strains JMY140K and JMY075, respectively. The results showed that the growth rate of JMY140K decreased with an increasing temperature (Figure 3a), and, interestingly, 40 °C is more conducive to the production of extracellular GABA (197.88 mg/L), as the growth rate at this temperature is 7.93 times higher than that at 30 °C (24.95 mg/L) (Figure 3b). For JMY075, the growth rate over 24 h decreased with an increasing temperature (Figure 3c), and the highest GABA production was reached at 37 °C (92.84 mg/L), at which point it was 2.61 times higher than at 25 °C (35.52 mg/L) (Figure 3d). The results showed that high-temperature stress could promote the production of extracellular GABA.

GABA can be naturally synthesized by lactic acid bacteria, which are potential probiotics widely present in fruits, vegetables, and fermented foods. *Lactobacillus brevis* GABA 100 showed higher production of GABA at 30 °C on the 12th day than that at 25 and 37 °C [28]. For *L. brevis* CRL 1942, higher GABA production (~255 mM) was observed at 30 °C for 48 h, which allowed the conversion of ~90% of the 270 mM MSG presented in the fermented medium [29]. *L. brevis* F064A isolated from Thai fermented sausage proved to be an excellent GABA producer, producing 2.85 ± 0.10 mg/mL of GABA and tolerating acidic conditions [30]. *L. paracasei* NFRI 7415 was also a GABA producer: it managed to significantly increase GABA production to 210 mM at an initial pH of 5.0 compared to that at pH 4.0 or pH 6.0 [31]. Compared to lactic acid bacteria, non-conventional yeasts may have superior stress resistance and serve as a novel alternative in GABA production.

#### 3.3.2. Growth Rates and GABA Production of Two Strains Under Different Levels of Acetic Acid or Ethanol Stress

**Figure 4:**
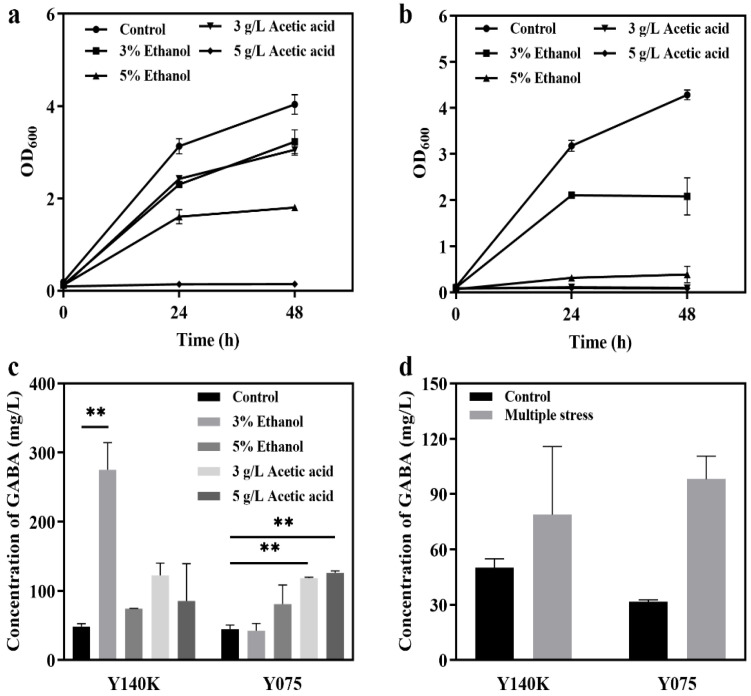
Growth and extracellular GABA production of *K. marxianus* Y140K and *M. reukaufii* Y075 under acetic acid and ethanol stress. (**a**,**b**) Growth of Y140K (**a**) and Y075 (**b**) in YPD liquid media supplemented with ethanol or acetic acid, respectively; (**c**) the concentrations of extracellular GABA produced by Y140K and Y075 in YPD liquid media after adding ethanol or acetic acid, respectively; (**d**) the concentrations of extracellular GABA produced by Y140K and Y075 in YPD liquid media after subjection to multiple stresses. The concentration of extracellular GABA produced by Y140K was detected at 48 h, and that for Y075 was detected at 24 h. Data are averages from two independent experiments; error bars represent SD. **, *p*-value < 0.01.

The strains were cultured under different levels of acetic acid or ethanol stress at 25 °C (JMY075) or 30 °C (JMY140K) to determine their growth rates and GABA production capacities. The ethanol concentrations were 3% and 5% (*v*/*v*), and the acetic acid concentrations were 3 g/L and 5 g/L, respectively. For strain JMY140K, the growth rate was affected by the addition of acetic acid and ethanol. Its growth rates were lower than those of the control. The decrease rate corresponded to an order of 3% ethanol = 3 g/L of acetic acid < 5% ethanol, and there was no growth after adding 5 g/L of acetic acid (Figure 4a). Under different stress additions, 3% ethanol most obviously promoted extracellular GABA production, which was 5.68 times (275.09 mg/L) higher than that for the control (48.40 mg/L), and under other conditions, the extracellular GABA production levels were 122.26 mg/L (3 g/L acetic acid), 85.48 mg/L(5 g/L acetic acid), and 74.36 mg/L (5% ethanol), all of which were higher than the control (Figure 4c). The above results show that the most favorable fermentation stresses for GABA production corresponded to the following order: 3% ethanol, 3 g/L of acetic acid, 5 g/L of acetic acid, and 5% ethanol, respectively.

It was shown that strain JMY075 had poor tolerance to acetic acid and ethanol, only growing weekly by adding 3% ethanol and barely growing by adding 5% ethanol, 3 g/L of acetic acid, and 5 g/L of acetic acid, respectively (Figure 4b). The addition of acetic acid promoted the production of extracellular GABA. After adding 3 g/L and 5 g/L of acetic acid, the concentrations of GABA were 118.30 mg/L and 125.94 mg/L, 2.65 and 2.82 times greater than the concentration of the control, respectively (Figure 4c). And the highest concentration of extracellular GABA obtained in this study is 35 times higher than that produced by a previously reported *K. marxianus* strain that uses glucose to produce GABA (7.78 mg/L) [17].

In addition, to study the effect of multiple stress conditions on the promotion of extracellular GABA production, 5 g/L of glutamic acid and 0.1 mM PLP (pyridoxal phosphate) were added to the basal fermentation medium. JMY140K was cultured at 30 °C with 3% ethanol; JMY075 was cultured at 37 °C with 3 g/L of acetic acid. The results indicate that multiple stress conditions could not better promote the production of extracellular GABA (Figure 4d).

### 3.4. Effects of Fermentation Stress Conditions on Intracellular GABA Production

**Figure 5:**
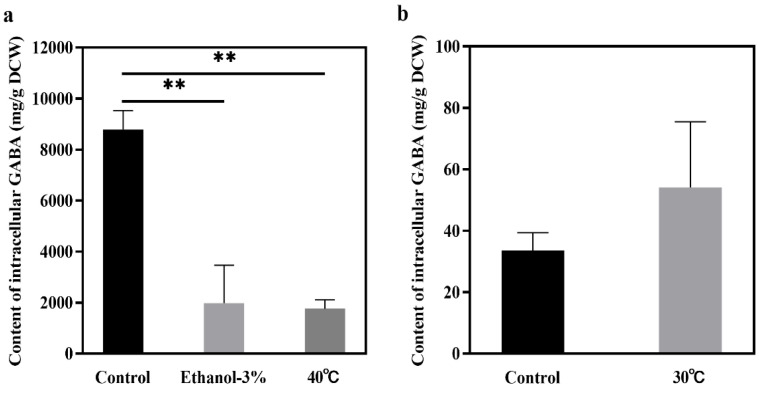
Intracellular content of GABA in *K. marxianus* Y140K and *M. reukaufii* Y075. (**a**) Intracellular content of GABA of Y140K at 48 h; (**b**) intracellular content of GABA of Y075 at 24 h. The control corresponds to non-stress conditions. Data are averages from two independent experiments; error bars represent SD. **, *p*-value < 0.01.

Previous studies have shown that GABA is selectively excreted outside the cell during metabolism and most of the GABA is stored inside the cell [32]. In this study, intracellular accumulation of GABA was detected under different fermentation stresses (Figure 5). For strain JMY140K, the level of intracellular GABA production was lower than the level of extracellular GABA production under stress conditions (Figure 5a), indicating that the stress conditions may be conducive to promoting GABA efflux or GABA synthesis and efflux. For strain JMY075, the level of intracellular GABA production was the same as that of the control, but the level of extracellular GABA production was higher than that of the control (Figure 3d), indicating that high-temperature stress amounting to 30 °C promoted the total synthesis of GABA. Under the conditions of 3% ethanol addition and 40 °C incubation, the intracellular GABA content of JMY140K was 1979.26 mg/g DCW and 1760.42 mg/g DCW, respectively. Under the condition of 30 °C incubation, the intracellular GABA content of JMY075 was 59.04 mg/g DCW (Figure 5b). In future industrial applications, intracellular and extracellular GABA can be extracted via high-temperature boiling, ethanol extraction, or cell wall disruption for comprehensive utilization.

### 3.5. Potential Mechanisms of the Effects of Stress Conditions on the Extracellular GABA Production Ability of JMY140K

#### 3.5.1. Detection of ROS and Cell Membrane Permeability Under Stress Conditions

**Figure 6:**
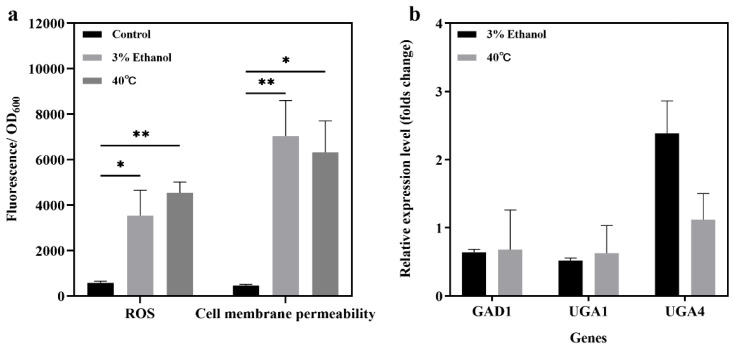
Mechanisms related to enhanced GABA production by *K. marxianus* Y140K under different stress conditions. (**a**) Intracellular ROS and cell membrane permeability of Y140K; data were detected at 48 h. (**b**) Transcription levels of the key genes in the GABA metabolism of Y140K under different conditions when compared to the control group. Data are averages from at least two independent experiments; error bars represent SD. *, *p*-value < 0.05; **, *p*-value < 0.01.

Intracellular ROS levels can increase under external stress conditions [33], allowing cells to produce stress-protective substances, such as GABA, to cope with the increase in intracellular ROS levels. The results obtained in this study indicate that external stress conditions may promote GABA efflux. Therefore, the relationships between GABA production, changes in intracellular ROS, and the cell membrane permeability of JMY140K were investigated in this study. The results showed that the levels of intracellular ROS and cell membrane permeability were significantly higher under the stress conditions of 3% ethanol and high temperatures, and the stimulation induced by a high temperature was better than that for 3% ethanol with respect to the increase in intracellular ROS levels but less than that induced by 3% ethanol with respect to cell membrane permeability (Figure 6a). Under the stress conditions tested in this research, combining the relationship between intracellular ROS, cell membrane permeability, and GABA production, the results indicated that there was no direct linear relationship between the stress conditions and GABA production. In this study, when 3% ethanol was added, the intracellular GABA content was significantly lower (Figure 5a), but extracellular GABA production was significantly higher (Figure 4c); under high-temperature conditions, extracellular GABA content increased (Figure 4c), while intracellular GABA content decreased (Figure 5a), and the intracellular ROS levels and cell membrane permeability were significantly higher (Figure 6a), indicating that the stress conditions increased cell membrane permeability, promoted the secretion of intracellular GABA outside of the cell, and increased the extracellular GABA content but did not increase total GABA production.

#### 3.5.2. Key Functional Genes’ Expression Under Stress Conditions

The expression levels of synthetic genes (GAD1) and catabolic genes (UGA1 and UGA2) in the GABA shunt pathway of *K. marxianus* are related to GABA production [15,34]. In *Candida glycerinogenes*, the expression of the UGA4 gene encoding GABA-specific permease is strongly induced by hypertonic stress. Under hypertonic conditions, exogenous GABA promotes intracellular GABA accumulation and cell growth, and overexpression of glutamate decarboxylase gene GAD1 can increase intracellular GABA content and promote cell growth under hypertonic conditions [35]. Metabolomics analysis of *Saccharomyces cerevisiae* revealed the key role of the GABA shunt in resisting various inhibitors, such as furfural, acetic acid, and phenol [36]. The GABA-producing glutamate decarboxylase GAD is required for normal oxidative stress [37]. Overexpression of the GAD1 gene increases cell tolerance to H_2_O_2_ and hydrazine. A study showed that the GABA shunt pathway plays a key role in limiting the production of ROIs (reactive oxygen intermediates) [38]. The GABA shunt pathway restricts the flow of carbon from α-ketoglutarate to succinic acid to limit the production of ROI, thereby protecting yeast cells from heat damage.

The results above show that the intracellular and extracellular GABA contents are different under different stress conditions. In this study, the expression levels of three genes, GAD1 (encoding the glutamic acid decarboxylation enzyme, for GABA synthesis), UGA1 (encoding GABA transaminase, for GABA degradation), and UGA4 (encoding the GABA transporter, for GABA absorption), in the GABA metabolism pathway of *K. marxianus* JMY140K were investigated under different stress conditions; the results are shown in Figure 6b.

In *K. marxianus* JMY140K, the expression of the UGA4 gene was upregulated to a more noticeable degree under the stress condition of 3% ethanol compared to high temperature, which further confirmed that under the stress condition of 3% ethanol, the UGA4 gene participated in intracellular GABA efflux, and the extracellular GABA content increased. The expression levels of the three genes under other conditions were not significantly different, and the expression levels were all downregulated, indicating that the activity of the GABA pathway was reduced under stress conditions (Figure 6b). This can be explained by the previously measured intracellular GABA content (Figure 5a). Under the conditions of 3% ethanol and 40 °C high-temperature stress, the intracellular GABA content was lower than that of the control, while the extracellular GABA content was higher than that of the control, indicating that the GABA production of the whole cell was not increased under stress conditions. Furthermore, combined with the increased intracellular ROS levels and cell membrane permeability under stress conditions (Figure 6a), the increase in extracellular GABA content may be the result of increased cell membrane permeability caused by stress conditions, resulting in greater GABA efflux. The downregulation of GAD1 gene expression may have occurred because cells are more inclined to synthesize other stress-protective substances or reduce the burden of cells to maintain life functions, thereby reducing the synthesis of GABA.

### 3.6. GABA Production of JMY140K Using Rice Straw Hydrolysate

**Figure 7:**
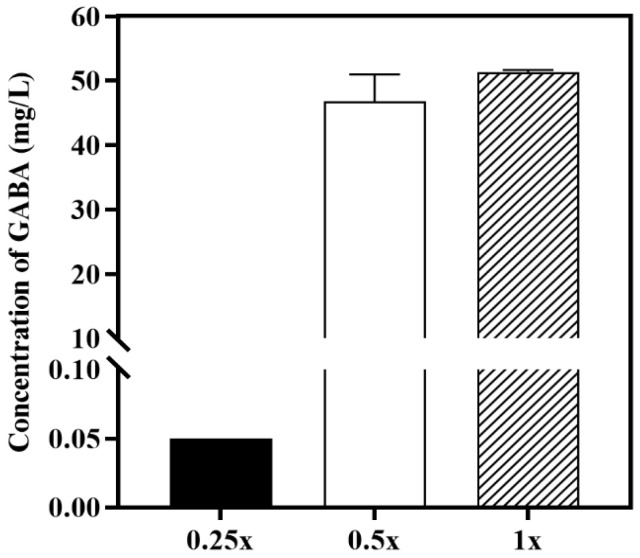
Extracellular GABA production by *K. marxianus* Y140K using rice straw hydrolysate. The concentration of extracellular GABA was detected at 48 h. The original hydrolysate (1×) and hydrolysates diluted 2 times (0.5×) and 4 times (0.25×) were used in this study. Data are averages from at least two independent experiments; error bars represent SD.

*K. marxianus* JMY140K can use rice straw hydrolysate to produce GABA. In this study, the original hydrolysate (0.25× in Figure 7) and the hydrolysate diluted 2 times (0.5× in Figure 7) and 4 times (1× in Figure 7) were used as a culture medium to detect GABA production. The results showed that extracellular GABA reached a level comparable to that of the YPD medium (Figure 7), indicating that rice straw hydrolysate can be used as a cheap fermentation medium substitute for GABA production. The results indicate that *K. marxianus* JMY140K was able to withstand the inhibitors produced in rice straw hydrolysate to produce GABA. Rice straw hydrolysate contains various inhibitors that negatively affect the growth of the yeasts, thereby repressing their ability to produce GABA. The results in this study confirm that a low concentration of rice straw hydrolysate leads to higher GABA production. Although industrial production of GABA using lignocellulosic biomass is still at an early stage, our results provide a basis for sustainable GABA production for potential future applications. *K. marxianus* is a food-grade yeast widely used in industrial food and biotechnological applications [39]. *K. marxianus* JMY140K will have a wider range of applications in industrial food and biotechnology.

### 3.7. Skincare Efficacy of JMY140K Filtrates

#### 3.7.1. The Proliferative Advantage of JMY140K Filtrates for Human Skin Cells

Human skin is a continuously self-renewing organ that dynamically manages the outside–inside–outside relationships of the human body and actively participates in host defense. It is essentially composed of two layers, namely, the epidermis on the outside and the dermis underneath, with keratinocytes representing 95% of the epidermal cells and FBs being the most abundant cells in the dermis. The effect of JMY140K filtrates on the proliferation of FB is shown in Table 1. Under the condition of 28 °C fermentation, the highest activity of FB cells reached 107.61% after adding 2% (*v*/*v*) of the filtrates of JMY140K fermented at 28 °C; but the highest activity of FB cells reached 156.85% after adding 5% of the filtrates of JMY140K fermented at 40 °C, a level significantly higher than that under the proliferation effect of the filtrates of JMY140K fermented at 28 °C.

The DP cells at the base of the hair follicle are a group of dermal mesenchymal cells. DP cells act as a signaling center for epithelial–mesenchymal cross-talk that regulates the balance between matrix cell proliferation and hair production, serving as an in vitro screening model for studying hair growth [40]. The results show that compared with the blank group, the filtrates of JMY140K within the range of 5% to 15% could effectively enhance the vitality of DP cells in a concentration-dependent manner, indicating that the filtrates of JMY140K have the potential to promote hair follicle growth and fertility. The highest activity of DP cells reached 135.49% after adding the filtrates of JMY140K fermented at 40 °C; this level is higher than that of 123.21% reached after adding the filtrates of JMY140K fermented at 28 °C. The details are shown in Table 2.

We found that the filtrates of JMY140K obtained from JMY140K can promote the proliferation of FBs and DP, indicating that the filtrates of JMY140K can maintain healthy skin and repair aging skin cells and have the potential to promote hair follicle growth and fertility. It is worth noting that the proliferation capacity of the filtrates of JMY140K fermented at 40 °C was significantly higher than that of the filtrates of JMY140K fermented at 28 °C. The metabolites from cells cultured at 28 °C and 40 °C were different, so besides the increased content of extracellular GABA, we cannot rule out the possibility that other substances may also have protective effects.

#### 3.7.2. Reparative Effect of JMY140K Filtrates on UV-Induced Cell Damage

UV radiation is divided into three major types by wavelength: UVA (315–400 nm), UVB (280–315 nm), and UVC (100–280 nm). Of these, UVA and UVB account for approximately 95% and 5%, respectively, of solar UV radiation, while UVC is filtered out by ozone [41]. UVA penetrates deep into the dermis and causes damage indirectly by forming reactive oxygen free radicals (ROS), while UVB reaches only the epidermis and causes direct DNA damage. UV radiation is known to cause skin aging and wrinkling, which is the primary cause of photoaging [42]. HaCaT is a long-lived, spontaneously immortalized human keratinocyte line that has been widely used as a suitable in vitro model for studies of skin biology and differentiation [43]. Using UVB to irradiate HaCaT and dermal FBs or applying simultaneous UVA + UVB irradiation to FB cells, a skin photo damage model was established to evaluate the protective effect of JMY140K filtrates with respect to UV damage to skin cells.

Compared with the irradiated group without samples, the addition of 5% (*v*/*v*) concentrations of the filtrates of JMY140K fermented at 28 °C and 40 °C, respectively, restored the viability of HaCaT cells irradiated with UVB to 100.52% and 127.92%, respectively (Table 3); the addition of 20% of the filtrates of JMY140K fermented at 28 °C and 40 °C, respectively, restored the viability of FB cells to 150.55% and 158.86%, respectively (Table S1), indicating that the filtrates of JMY140K also have a significant protective effect on UV-damaged FB cells, and the repair effect of the filtrates of JMY140K fermented at 40 °C was significantly better than that of the filtrates of JMY140K fermented at 28 °C. The detailed results are shown in Table 3 and Table S1.

We found that the filtrates of JMY140K can promote the repair of HaCaT cells and FB cells, indicating that the filtrates of JMY140K have the potential to repair the damage induced by UV and probably prevent the accumulation of oxidative damage that leads to UV-induced photoaging. And the proliferation of the filtrates of JMY140K fermented at 40 °C was significantly higher than that of the filtrates of JMY140K fermented at 28 °C.

#### 3.7.3. Effects of Different Filtrates of JMY140K on Intracellular ATP in Human Skin Cells

Cells require energy to carry out the reactions that maintain their viability, growth, and proper function [44]. The dominant chemical energy currency in cells is ATP. Previous studies have shown that decreased ATP levels in skin cells can lead to damage caused by environmental factors such as skin aging and ultraviolet radiation [45]. Therefore, maintaining adequate intracellular ATP levels is important for the overall health and function of skin cells. Based on this, the filtrates of JMY140K used in this study may have the potential to maintain the overall health and function of skin cells.

The detailed results are shown in Tables S2 and S3, which indicate that the filtrates of JMY140K fermented at 28 °C and 40 °C can effectively increase the relative level of ATP in cells. Under the fermentation condition of 28 °C, the highest activities of HaCaT, FBs, and DP ranged up to 109.80%, 120.71%, and 118.42%, respectively. Under fermentation conditions of 40 °C, the highest activities of HaCaT, FBs, and DP ranged up to 118.67%, 123.28%, and 123.23%, respectively. The highest relative ATP content of the filtrates of JMY140K fermented at 40 °C was higher than that of the filtrates of JMY140K fermented at 28 °C, indicating that filtrates of JMY140K fermented at 28 °C and 40 °C can effectively increase the relative level of ATP in cells. In particular, the effect of the filtrates of JMY140K fermented at a stress temperature of 40 °C on intracellular ATP was significantly better than that of the filtrates of JMY140K fermented at 28 °C. The results indicate that strain JMY140K can respond to external stress environments, granting the fermentation filtrate stronger skin care effects.

## 4. Conclusions

*K. marxianus* JMY140K and *M. reukaufii* JMY075 were found to be GABA producers. It was found that the intracellular and extracellular GABA production of the two non-conventional yeasts were both affected by the external stress conditions, including high temperatures and the addition of ethanol and acetic acid. *K. marxianus* JMY140K can utilize rice straw hydrolysate to produce GABA, which could be used as a cheap fermentation medium in industrial production. We also found that the filtrates of JMY140K can maintain a healthy condition of human skin cells, and the bioactivities of the filtrates of JMY140K fermented at 40 °C were significantly better than those of the filtrates of JMY140K fermented at 28 °C. This study provides an alternative strategy for inducing the production of GABA by using appropriate stress conditions, which is beneficial for the sustainable production of other valuable bioactive agents.

## Figures and Tables

**Table 1:** Effect of fermentation filtrate (FF) of *K. marxianus* Y140K on the proliferation of fibroblast cells *.

Samples	Final Concentration	Relative Viability of Fibroblasts (%)	
		28 °C	40 °C
No-addition control (NT)	/	100.00 ± 7.34	
1% FF	0.1 mg/mL	106.09 ± 5.16	131.67 ± 8.78
2% FF	0.2 mg/mL	107.61 ± 8.37	130.84 ± 5.94
5% FF	0.5 mg/mL	103.18 ± 2.97	156.85 ± 6.02
10% FF	1.0 mg/mL	86.45 ± 7.45	145.50 ± 10.50

* 28 °C and 40 °C indicate fermentation filtrates (FFs) of *K. marxianus* Y140K, which was obtained from cultures at 28 °C and 40 °C, respectively. Relative viability of the fibroblast cells was calculated in comparison with that of the non-addition control.

**Table 2:** Effect of fermentation filtrate (FF) of *K. marxianus* Y140K on the proliferation of DP cells *.

Samples	Final Concentration	Relative Viability of DP Cells (%)	
		28 °C	40 °C
NT	/	100.00 ± 5.81	
5% FF	0.5 mg/mL	113.98 ± 9.77	122.24 ± 9.56
10% FF	1.0 mg/mL	116.01 ± 9.25	130.03 ± 10.44
15% FF	1.5 mg/mL	123.21 ± 13.91	135.49 ± 8.97

* 28 °C and 40 °C indicate fermentation filtrate (FF) of *K. marxianus* Y140K from the cultures at 28 °C and 40 °C, respectively. Relative viability of the human scalp dermal papilla (DP) cells was calculated in comparison with that of the non-addition control.

**Table 3:** UV damage and repair effect of fermentation filtrate (FF) of *K. marxianus* Y140K on HaCaT cells *.

Samples	Final Concentration	Relative Viability HaCaT (%)	
		28 °C	40 °C
NT	/	100.00 ± 6.37	
2% FF	0.2 mg/mL	79.71 ± 6.64	104.22 ± 3.03
5% FF	0.5 mg/mL	100.52 ± 5.97	127.92 ± 8.01
10% FF	1.0 mg/mL	72.93 ± 9.84	103.42 ± 3.14

* 28 °C and 40 °C indicate fermentation filtrate of *K. marxianus* Y140K (FF) obtained from the cultures at 28 °C and 40 °C, respectively. Relative viability of the human epidermal (HaCaT) cells was calculated in comparison with that of the no-addition control.

## Data Availability

The original contributions presented in the study are included in the article/Supplementary Material, further inquiries can be directed to the corresponding authors.

## Supplementary Materials

The following supporting information can be downloaded at https://www.mdpi.com/article/10.3390/jof11010020/s1. Table S1: UV damage and repair effect of fermentation filtrate (FF) of *K. marxianus* Y140K on FB cells; Table S2: Effects of fermentation filtrate (FF) of *K. marxianus* Y140K on intracellular ATP levels in HaCaT cells; Table S3: Effects of fermentation filtrate (FF) of *K. marxianus* Y140K on ATP levels in FB cells and DP cells.
