# Supplementary material for: Stress-Driven Production of γ-Aminobutyric Acid Using Non-Conventional Yeast Strains Kluyveromyces marxianus JMY140K and Metschnikowia reukaufii JMY075
Source: J Fungi (Basel). 2024 Dec 31;11(1):20. doi: 10.3390/jof11010020 (PMC11766319; doi:10.3390/jof11010020)
Supplement: Supplementary file 1 [file jof-11-00020-s001.zip › jof-3351531-supplementary.pdf]

**Table S1** UV damage and repair effect of fermentation filtrate (FF) of *K. marxianus* Y140K on FB cells*

| Samples | Final concentration | Relative viability of FB cells (%) | |
|---|---|---|---|
| | | 28°C | 40°C |
| NT | / | 100.00±13.46 | |
| 2.5% FF | 0.25 mg/mL | 135.23±7.27 | 138.73±10.72 |
| 20% FF | 2.0 mg/mL | 150.55±4.85 | 158.86±2.02 |

*28°C and 40°C indicates fermentation filtrate (FF) of *K. marxianus* Y140K obtained from the cultures at 28°C and 40°C, respectively. Relative viability of fibroblast (FB) cells was calculated comparing with that of the non-addition control.

**Table S2** Effects of fermentation filtrate (FF) of *K. marxianus* Y140K on intracellular ATP levels in HaCaT cells*

| Samples | Final concentration | ATP relative content (%) | |
|---|---|---|---|
| | | 28°C | 40°C |
| NT | / | 100.00±5.50 | |
| 1%FF | 0.1 mg/mL | 91.07±5.68 | 112.88±3.68 |
| 2%FF | 0.2 mg/mL | 101.39±5.98 | 111.07±2.36 |
| 5%FF | 0.5 mg/mL | 98.13±7.55 | 118.67±4.96 |
| 10%FF | 1.0 mg/mL | 109.80±3.94 | 114.49±4.36 |

*28°C and 40°C indicates fermentation filtrate (FF)of *K. marxianus* Y140K obtained from teh cultures at 28°C and 40°C, respectively. ATP relative content of the human epidermal (HaCaT) cells was calculated comparing with that of the non-addition control.

**Table S3** Effects of fermentation filtrate (FF) of *K. marxianus* Y140K on ATP levels in FB cells and DP cells*

| Samples | Final concentration | ATP relative content (%) in FB cells | | ATP relative content (%) in DP cells | |
|---|---|---|---|---|---|
| | | 28°C | 40°C | 28°C | 40°C |
| NT | / | 100.00±5.40 | | 100.00±3.27 | |
| 5% FF | 0.5 mg/mL | 101.83±3.57 | 117.62±5.97 | 113.18±3.60 | 116.38±4.31 |
| 10% FF | 1.0 mg/mL | 111.48±3.45 | 117.97±3.10 | 117.16±1.77 | 119.97±1.83 |
| 15% FF | 1.5 mg/mL | 112.59±7.62 | 120.35±3.48 | 116.49±2.72 | 122.30±2.33 |
| 20% FF | 2.0 mg/mL | 120.71±2.58 | 123.28±4.93 | 118.42±3.20 | 123.23±2.73 |

*28°C and 40°C indicates fermentation filtrate (FF) of *K. marxianus* Y140K from the cultures at 28°C and 40°C, respectively. ATP relative content of the fibroblast (FB) cells and the human scalp dermal papilla (DP) cells was calculated comparing with that of the non-addition control.
